# Optimizing Evaluation of Older Adults With Vision and/or Hearing Loss Using the interRAI Community Health Assessment and Deafblind Supplement

**DOI:** 10.3389/fresc.2021.764022

**Published:** 2021-12-13

**Authors:** Andrea Urqueta Alfaro, Cathy McGraw, Dawn M. Guthrie, Walter Wittich

**Affiliations:** ^1^School of Optometry, Université de Montréal, Montréal, QC, Canada; ^2^Centre de Recherche Interdisciplinaire en Réadaptation du Montréal métropolitain/Centre de réadaptation Lethbridge-Layton-Mackay du Centres Intégrés Universitaires de Santé et de Services Sociaux du Centre-Ouest-de-l'Île-de-Montréal, Montréal, QC, Canada; ^3^Centre de Recherche Interdisciplinaire en Réadaptation du Montréal métropolitain/Institut Nazareth et Louis-Braille du Centres Intégrés de Santé et de Services Sociaux de la Montérégie-Centre, Longueuil, QC, Canada; ^4^Department of Kinesiology and Physical Education, Wilfrid Laurier University, Waterloo, ON, Canada

**Keywords:** deafblindness, hearing loss, vision loss, low vision, hard of hearing, dual sensory impairment (DSI), screening, interRAI

## Abstract

**Purpose:** Service providers must identify and assess older adults who have concurrent vision and hearing loss, or dual sensory impairment (DSI). An assessment tool suitable for this purpose is the interRAI Community Health Assessment (CHA) and its Deafblind Supplement. This study's goal was to explore this assessment's administration process and to generate suggestions for assessors to help them optimize data collection.

**Methods:** A social worker with experience working with adults who have sensory loss, who was also naïve to the interRAI CHA, administered the assessment with 200 older adults (65+) who had visual and/or hearing loss. The assessor evaluated the utility of the instrument for clinical purposes, focusing on sections relevant to identifying/characterizing adults with DSI.

**Results:** Suggestions include the recommendation to ask additional questions regarding the person's functional abilities. This will help assessors deepen their understanding of the person's sensory status. Recommendations are also provided regarding sensory impairments and rehabilitation, in a general sense, to help assessors administer the interRAI CHA.

**Conclusions:** Suggestions will help assessors to deepen their knowledge about sensory loss and comprehensively understand the assessment's questions, thereby allowing them to optimize the assessment process and increase their awareness of sensory loss in older adults.

## Introduction

There is an ongoing rise in the prevalence of dual sensory impairment (DSI), defined as the combination of any level of concurrent vision and hearing loss irrespective of age of onset (1). DSI interferes with a person's ability to communicate, acquire information, and perform daily activities (2). The prevalence of DSI increases with increasing age, and estimates vary among diverse sub-groups of older adults (65+), ranging from 20% in residential care or day centers (3) to 38% among centenarians (4). Most developed countries are experiencing population aging, with the most rapid increase among people aged 85+ years, who are also the most likely to experience DSI and its associated challenges (5, 6). Given the correlation between aging and sensory impairment, it is estimated that as many as one in three individuals over the age of 50 have either reduced vision, impaired hearing, or DSI (1). Once detected, rehabilitation services for sensory loss are available, such as those in Montreal, Canada, where 69% of clients receiving services for DSI are over the age of 64 (7).

As a result of population aging, the proportion of older adults who access any type of health service in developed countries is currently high and will likely increase over the coming decades. For example, in 2009–2010 in Canada, seniors made up 14% of the population, but accounted for 40% of acute hospital stays (8). The detection and evaluation of sensory impairments are of the utmost importance because several aspects of health care delivery, and communication with health professionals, depend on it. However, many first-line health care providers operate under the basic assumption that their clients can hear and see them; however, in a third of their older patients this may not always be the case (1). Therefore, it is critical to provide first-line health service providers with standardized and user-friendly assessment tools to identify individuals with DSI, assess their needs and strengths, and guide the design of rehabilitation strategies.

The interRAI Community Health Assessment (CHA) and its Deafblind Supplement (DbS) is a standardized, valid, and reliable assessment instrument for use with adults (18+), including those with DSI (9, 10). This assessment was created by interRAI (http://www.interrai.org/), a not-for-profit research network of 90 members from 35 countries, who have a mandate to develop and test assessment systems that aim to improve the quality of life and delivery of services for vulnerable populations, including older persons and those with disabilities. The interRAI instruments are based on significant input from content experts, clinicians and service providers, are used internationally, including being mandated in several regions in Canada, and are backed by studies evaluating their psychometric properties (11). The goal of the instrument is to help an assessor identify the needs and abilities of adults who reside in the community and to guide the assessor in terms of developing a service plan.

The interRAI CHA consists of roughly 150 items, and four supplemental assessments, one of which is the DbS. The responses to two items on the CHA that refer to the person's functional vision and hearing, will “trigger” whether or not the person likely has DSI and should therefore be assessed more comprehensively with the DbS. The DbS includes an additional 150 items that capture further detail about the person's vision and hearing and other issues relevant for an individual with DSI (e.g., age of onset of sensory loss, diagnoses, communication, psychosocial well-being) (9). Research shows the assessment has good internal consistency and convergent validity (12), as well as some preliminary evidence of acceptable inter-rater reliability (13). The assessment constitutes a comprehensive assessment of a person's overall health and functional abilities, as well as a more specific evaluation of domains that are relevant in individuals with DSI. A person is classified as having DSI if they report having at least minimal difficulty with near vision activities (using vision device if applicable) and with hearing (14). The assessor uses the gathered information and his/her own clinical judgement to decide how to proceed in terms of referrals (e.g., referral to programs/resources specific to persons with DSI) and implementation of a service plan.

While many age-related conditions have been investigated, few studies have examined the assessment and service needs of older persons with DSI (15, 16). After completion of the data collection for a larger protocol on sensory-cognitive aging (17, 18), the team members noticed during team discussions that the individual who had collected the data had developed a unique expertise and extensive case notes that would allow the team to develop recommendations to improve the administration of the interRAI CHA and its DbS. Based on this expertise, the aim of the present study was to develop suggestions that can help future assessors optimize data collection with the interRAI CHA and DbS. This study utilized the experience of a knowledgeable assessor, who administered the interRAI CHA and DbS to 200 older adults (61+) with sensory impairments, to qualitatively evaluate the instrument and provide recommendations that would be useful to others completing the assessment who may be less familiar with the nuances of communication and interviewing older persons with sensory challenges. This evaluation was undertaken with the needs of three groups of stakeholders in mind: 1. persons who experience significant functional limitations due to either a single or dual hearing and/or vision loss; 2. assessors new to the instrument; and 3. those who are experienced in developing and using interRAI tools. As much as any assessment tool can be useful in understanding the needs and preferences of the person, it should be recognized that an assessment should always be considered as a compliment to clinical judgement, and not a replacement, and that all assessments are inherently imperfect. This paper presents the resulting recommendations and aims to improve assessor's awareness of sensory loss in older adults and how it impacts the assessment process in order to facilitate assessors' administration of the instrument and optimize the quality of the information captured.

## Materials and Equipment

This study required the use of the interRAI community health (CHA) assessment form and user's manual (10).

## Methods

The interRAI CHA is generally administrated with a global focus on multiple aspects of functioning (19). Sensory health only becomes the focus once the DbS has been triggered during the administration of the CHA; therefore, it has been the clinical experience of our team members that individuals who administer the CHA do not automatically consider potential administration barriers (e.g., subtle cues indicating the need for communication accommodations) that may be present before a sensory impairment has been detected or identified. The awareness of such barriers may be largely connected to the level of clinical experience of the administrator, and at present there are no clear training recommendations or administration considerations in place. Most individuals who administered the interRAI CHA therefore do not have much experience with the assessment of persons living with vision and/or hearing impairment. While conducting a series of studies on sensory-cognitive aging using the interRAI CHA (17, 18), our team realized that our assessor combined a list of rare qualities that make her unique as an administrator of the interRAI tool.

### The Expert Participant

The assessor is a social worker with over 30 years of direct clinical experience in the context of sensory rehabilitation, and has developed and implemented a sensory screening program (13, 20). Her work experience and her professional interactions with sensory rehabilitation professionals allowed her to develop extensive expertise on how to interact and communicate with persons living with different levels of sensory impairment., and how to explore and assess their functional abilities and needs. She participated in a two-day education session on how to administer the assessment based on the information available in the manual produced by interRAI. This included instructions for obtaining information from secondary sources, how to enter the data into the software system and how to interpret information generated by the software (e.g., scores on the health index scales). While the social worker had no prior experience administering the interRAI assessments, she had extensive interviewing skills, clinical experience and knowledge in the field of sensory rehabilitation who, by the end of the data collection period, also may be the first person ever to have administered the interrail CHA and its DbS to 200 older adults with confirmed sensory impairments. These characteristics led the team to consider her a “deviant case,” given her exceptional skill set and professional placement. The idea to synthesize the recommendations presented here did not emerge until after data collection was complete. Therefore, she and the team were unaware of the purpose of the present study, because its idea emerged afterwards.

### Participants

To be included in this study, participants had to be eligible for sensory rehabilitation services as defined by the Quebec Ministry of Health, with a visual acuity in the better eye with best correction of 20/60 (6/18) or less, or a visual field diameter of <60 degrees, or hemianopsia (loss of half of the visual field); and/or an unassisted pure tone average decibel hearing level (dB HL) in the better ear of 35 dB HL or more (21, 22). Additionally, it was required that participants had an initial evaluation by a sensory rehabilitation center at least 6 months prior to data collection, and had received sensory rehabilitation within the past 3 years. This excluded individuals who had recently undergone extensive intake/initial interviews by their respective rehabilitation centers. Study exclusion criteria included persons unable to communicate in English or French, those unable to communicate verbally, and clients who could not be reached by phone. All study procedures were reviewed and approved by the *Centre de recherche interdisciplinaire en réadaptation de Montréal métropolitain* (CRIR-1018-1114).

Participants were recruited through the respective programs of three Québec sensory rehabilitation establishments: (1) Lethbridge-Layton-Mackay Rehabilitation Centre (LLM); (2) Institut Nazareth et Louis-Braille (INLB); and (3) Institut Raymond-Dewar (IRD). Included within LLM are persons with visual impairment that participate in the Day Center, 60% of whom have compromised hearing (23). Some participants, who attended an INLB and IRD inter-establishment program (*programme surdicécité*), were registered in both of those centers. Participants were recruited by staff from each of the three rehabilitation centers. The staff provided eligible participants with information concerning the nature and voluntary participation in the study and gave them an opportunity to ask questions. Upon receipt of their verbal consent, their names were forwarded to this study's assessor (CM), who was responsible for scheduling the interview. After receiving more details about the study protocol, 21 (INLB = 10, IRD = 6, LLM = 5) of the 221 persons who had initially agreed to participate, canceled their participation in the study before data collection began, mentioning reasons related to health issues, as well as limited energy.

The final sample consisted of 200 adults aged 61 and over (61%women, 39% men) with a mean age of 81.3 years. Based on the interRAI CHA and DbS, 35% of the sample had only hearing loss (hard of hearing or HH), 29% had only visual loss (visually impaired or VI), and 37% had DSI. Among participants with VI solely, whose medical records contained visual acuity measurements (*N* = 120), the best eye's average distance visual acuity in logMAR was 0.9. Among participants that were solely HH, whose medical records included hearing loss measurements (*N* = 93), the best ear's average hearing was 59 dB HL. Additional details about the sensory and cognitive profiles of these participants has been presented elsewhere (17, 18). Please note that the process of telephone recruitment prohibited us from recruiting individuals who primarily communicated using sign language, or those whose hearing loss was too profound for verbal communication. Even though complete blindness was not an exclusion criteria, none of the recruited participants utilized braille for print access. Of the participants who were assessed with the Montreal Cognitive Assessment (MoCA; see “Measures” for a detailed description), most passed this test (56% of *N* = 198), indicating they were not at risk for mild cognitive impairment (24). Of the two participants that were not assessed with the MoCA, one died before the scheduled appointment and, in the other case, it was not administered because the assessor felt that the test would have caused the participant undue psychological stress.

### Measures

#### InterRAI CHA and DbS

The roughly 150 items in the interRAI CHA capture basic demographic information about the person and detailed information across 13 domains, including abilities in activities of daily living (e.g., bathing, dressing, grooming), instrumental activities of daily living (e.g., using the telephone, managing finances), cognition, social functioning, mental health, pain, hearing, and vision. Once the CHA is completed electronically, a series of health index scales can be automatically generated. For example, the Deafblind Severity Index is based on two items within the CHA which measure functional vision and hearing. The Deafblind Severity Index ranges from 0 (no impairment in either sense) to 5 (severe impairment in both senses); a score of 3+ identifies individuals with any level of DSI (12). If an individual scores three or higher on the CHA, it is recommended that the assessor complete the DbS. This supplemental assessment includes ~150 items to gather further information across 11 domains considered relevant for the assessment of individuals with DSI, including vision and hearing (e.g., age of onset of vision/hearing loss, diagnoses, visual acuity and field diameter, alerting to sounds, devices used), communication (e.g., communication modes used, ability to communicate with family members), mood and behavior, level and type of informal support from friends/family, psychosocial well-being, and orientation and mobility/O&M (e.g., ability to move about in both familiar and unfamiliar environments). Based on previously published information (12), Table 1 provides an overview of the content captured within the interRAI CHA and DbS. All of the responses to items within the interRAI CHA and DbS are closed-ended; most are scored as yes/no, others are scored on an ordinal scale (typically 0–5, but sometimes up to 8). The typical time frame for assessment is the previous 3 days; a few items ask about the past 90 days[10].

**Table 1:** Key domains of the interRAI Community Health Assessment (CHA) and deafblind supplement.

**Domain**	**Number of items**	**Sample items**
**interRAI CHA section:**		
A. Identification	12	Birthdate, sex, living arrangement
B. Intake/Initial history	8	Residential history
C. Cognition	3	Decision making, memory or recall abilities
D. Communication and vision	4	Expression, comprehension, hearing/vision
E. Mood	12	Anger, withdrawal
F. Psychosocial well-being	10	Social relationships, length of time alone
G. Functional status	20	Instrumental/activities of daily living
H. Continence	1	Bladder continence
I. Disease diagnosis	19	Musculoskeletal, neurological, cardiac
J. Health conditions	25	Falls, fatigue, pain, tobacco/alcohol
K. Nutritional status	4	Weight loss, dehydration
L. Medications	12	List of medication, dose, unit, frequency
M. Treatment and procedures	11	Blood pressure, dental, hearing exam
N. Social supports	1	Relationship with family
O. Environmental assessment	1	Finances
P. Discharge	2	Last day of stay, living status after discharge
Q. Assessment information	2	Signature, date signed as complete
**Deafblind supplement section:**		
A. Identification	8	Name, health card number
B. Vision and hearing	50	Diagnosis, onset, stability, senses
C. Communication	15	Communication modes used daily
D. Education and employment	4	Education completed, employment status
E. Mood and behavior	6	Episodes of panic, social behavior
F. Informal support	22	Informal helper status, number of hours
G. Activities, involvement and psychosocial well-being	23	Preferred activities, sense of involvement
H. Functional status	2	Activities of daily living self-performance
I. Orientation and mobility	7	Aware of surroundings, ability to travel
J. Nutritional status	1	Mode of nutritional intake
K. Service utilization	21	Formal services, social interactions
L. Environmental assessment	4	Disrepair of the home, limited access
M. Assessment information	2	Signature, date signed as complete

The interRAI CHA and DbS is a copyrighted tool. Further information is available in the assessment's manual [10], which is available for purchase from interRAI (http://www.interrai.org/instruments/).

#### Administration of the InterRAI CHA and DbS

The assessor secured participants' written consent and provided them with a description and basic guidelines concerning the assessment process that included the topics to be covered, the interview format, and the respondents' ability to ask clarifying questions and take breaks. Participants chose the location where the interview was conducted. Most participants (61%) chose their homes, the remaining participants preferred an office appointment at their respective rehabilitation center (LLM = 38.5%, IRD = 0.5%). At the interview, participants were asked if they understood or had questions before proceeding. To meet the participant's individualized needs, the assessor used effective communication strategies, in surroundings suitable for clear two-way communication (25).

In scoring the assessment items, the assessor considered both the participants' answers and information from participants' medical records. Study team members had access to participants' files from their respective rehabilitation centers. From the files, demographic (e.g., sex, age) and health information (e.g., vision and/or hearing diagnoses) was obtained. Assessment responses were entered into a software system, using unique identifiers to ensure confidentiality. Interviews lasted ~90 min, and were conducted in the participant's service language, either English or French. To facilitate communication, a personal amplification device (Pocket Talker®, WilliamsAV, Eden Prairie, MN, USA), was made available to participants who required an assistive listening device. Similarly, a large-print version of the consent form was available, as needed. As part of her process as a rehabilitation professional, the assessor took detailed case notes on the administration of each assessment.

#### Montreal Cognitive Assessment

To obtain additional information about participants' cognitive status, the MoCA, a 10-min cognitive screening tool for identifying individuals as risk for mild cognitive impairment, was completed. Cognitive screening of participants was included in the protocol because the data contributed to a larger overall protocol on sensory-cognitive aging (17, 18). Mild Cognitive Impairment is a clinical diagnosis characterized by cognitive decline that goes beyond normal cognitive aging and in many cases leads to dementia (24). The tasks included in the MoCA assess attention, concentration, working memory, short-term memory, visuospatial abilities, executive functions, language, and orientation to time and place [see Nasreddine et al. (24) for further detail]. The original version of the MoCA (hereafter “full” MoCA) was developed for and validated in adults without sensory impairment (24). A version for persons with VI (hereafter “blind” MoCA) was created by eliminating the first 3 items of the scale that require vision (i.e., viewing, copying and, creating drawings) and adjusting the cut-off scores accordingly. Compared to the full MoCA, the blind version has higher specificity for detecting healthy participants and lower sensitivity for detecting those with Mild Cognitive Impairment (26).

Participants who had sufficient vision, based on self-report and/or the assessor's clinical opinion, completed the full MoCA; those with VI completed the blind MoCA. The MoCA was typically administered during the interview when the interRAI CHA and DbS were completed, with the exception of 5 participants who completed the MoCA at a second session.

### Qualitative Analysis

Given the unique professional experience of the assessor and her unusual experience of administering 200 interRAI CHA assessments to individuals with objectively confirmed sensory impairment, we explored different analytical approaches in order to frame our analyses. We were inspired by deviant case analysis (27), even though originally this approach was intended to explore whether an existing theory can explain extreme observations or whether a theory needs to be expanded (28). Here, we treat the skill set of our expert assessor as unique or extreme, given her rare combination of abilities and experiences. We therefore decided to explore her perspective on the administration of the interRAI CHA and its DbS with the goal of expanding the training guidelines for assessment administrators that have less experience and/or exposure to individuals with sensory impairments.

Based on the available detailed case notes, the assessor reflected on her experience conducting the interRAI CHA and DbS interviews with the goal of generating recommendations relevant to future assessors completing the instrument, and suggestions for edits to the instruments and manual. She then engaged in dialogue with the team members about these experiences, where by all team members brought their expertise on sensory impairment, assessment administration and the interRAI measures into the discussion. As a team, we developed the content of the tables in this manuscript, using a reflective and iterative process. Within the CHA, this process focused on the section “Communication and Vision” because two of its items are used to calculate the Deafblind Severity Index which in turn triggers the DbS. Within the DbS, we focused on three sections that included items related to vision and hearing, communication, and orientation and mobility (O&M). These domains were chosen because they provide information that is the most specific to people with DSI. Communication, either expressive or receptive, relies on hearing of speech and seeing of facial gestures and body movements and creates significant difficulty for individuals with DSI. O&M refers to strategies and devices that are designed for people with visual and/or hearing loss. As part of the synthesis and team discussion process, the assessor also consulted with other professionals in her program that work with the participants in this study with DSI, specifically a low vision optometrist, an audiologist, and an O&M specialist. These professionals provided feedback on the instrument's questions, and the assessor's suggestions to facilitate the its administration. After this process, the initial list of suggestions was finalized by the team members. Because the qualitative analysis did not reveal any suggestions that differed depending on the study participant's sex, and the lack of evidence indicating that the use of the interRAI CHA and DbS are influenced by a respondent's sex, no sex-based analyses were conducted.

## Results

Overall, the qualitative evaluation of the utility of the interRAI CHA and DbS generated suggestions that stress how vision and hearing include several abilities, all of which are worth exploring when assessing a person's visual and hearing functioning. If the assessor focuses on only one sensory ability, for instance only near vision but not at a distance, s/he will have an incomplete view of the respondent's sensory capacities or miss a sensory impairment altogether. Suggestions also chiefly highlight the fact that a person's specific sensory function can be more or less impaired depending on contextual factors that may not be present during the interview; for instance, hearing speech in a quiet vs. a noisy environment. Determining a respondent's sensory performance must include observations of the person's behavior but also the context of the observation, and will benefit from gaining information about the respondent's performance in a variety of settings. Lastly, this study brings attention to specific information on sensory impairment/rehabilitation that will aid assessors in the administration of the assessment, particularly those naïve to the field of sensory loss.

An overview of the suggestions is presented below. Then in Tables 2–5, each specific suggestion for the assessor is listed, in connection to its corresponding question in the assessment instrument. Please note that these suggestions are based on the exper assessor's own experience when administering the interRAI CHA and its deafblind supplement, or specifically address potential concerns she would anticipate with less experienced administrators that are unfamiliar with individuals living with sensory impairment.

**Table 2:** Supplemental guidelines for completing interRAI CHA section D communication and vision.

Specific item in the interRAI CHA (^*^)	**A. Suggestions for the assessor**	**B. Suggestions for the selection of the appropriate response option**
D1. Making self understood (Expression)	- It is important to remember that “difficulties finding words” does not refer solely to speech, but also to writing, sign language, and gestures, among other methods of communication. - Given that difficulties with expression rarely present as an isolated disability, the assessor may need to investigate causes other than hearing loss. The assessor should not assume that a respondent's difficulties with verbal expression are due to hearing loss, as they could also be the result of a cognitive impairment.	- Differentiating between the response options on the assessment of “usually” and “often” can be confusing. Some respondents may use these terms interchangeably; others may consider “Often” to signify more frequent than “Usually.” If the assessor has difficulty in choosing one over the other, they should remember that the difference between the two terms relies on whether the person requires prompting.
D2. Ability to understand others (Comprehension)	- A person's understanding is greatly impacted by adverse listening conditions (e.g., multiple conversations, background radio), and by the types of communication strategies used (e.g., face-to-face vs. at a distance). The assessor should not determine the comprehension level based solely on the respondent's performance during the interview. The interview may be conducted in the most optimal conditions for comprehension, which if considered alone, would overestimate the respondent's comprehension under controlled/regular circumstances. The assessor should ask about the respondent's ability to understand others under different contexts which present diverse challenges to comprehension. - The assessor may benefit from training on effective communication strategies for interacting with persons with hearing loss.	- It is important to differentiate between a person's comprehension vs. hearing. Comprehension involves the discrimination of speech and understanding verbal information, whereas hearing involves the detection of sounds. The assessor should remember that this item is strictly coding the person's comprehension and not hearing. A person may hear the assessor's verbal sounds but not fully discriminate speech, which will hinder comprehension. - As above, difficulties may present when using the code “usually” and “often.” If difficulties present, the assessor should emphasize that the difference between the two terms, depends on whether the person requires prompting.
D3. Ability to hear (with hearing appliance normally used)	- In the interRAI manual, most examples for hearing refer to the comprehension of verbal communication. However, the assessor should remember that this item evaluates the detection of all types of sounds, as opposed to the comprehension of verbal communication, which is assessed in item D2. - Environmental factors have a significant impact on a person's hearing. Even if the respondent reports having no difficulty understanding a normal conversation with hearing aid(s), the assessor should ask whether this is the case in both optimal and adverse listening conditions. The assessor should not determine the hearing level based solely on the respondent's performance during the interview. This interview may be conducted in the most optimal conditions for hearing, and thus when considered alone, may overestimate the respondent's hearing under regular circumstances. The assessor should ask the respondent about different real life contexts that present diverse challenges for hearing. - A common misperception of hearing aids is that they restore hearing to normal-to-near normal levels. Similarly, when only one hearing aid is worn, it is often assumed that hearing is normal in the unaided ear, while often times the unaided ear has worse hearing.	- The item response options corresponding to “moderate difficulty" and “severe difficulty” use the same labels as those commonly used for the diagnoses of hearing loss (HL) based on dB levels (i.e., Moderate: 41–55 dB HL, Severe: 71–90 dB HL). The assessor should be cautious about not interpreting these response option as equivalent to these diagnostic terms. - It is important for the assessor to base their decision on medical records as well, if available.
D4. Ability to see in adequate light (with glasses or with other visual appliance normally used)	- The questions in the manual are focused on reading print at near distance. However, vision loss can express as difficulties performing other visual functions besides reading up close. For instance, a person who has a visual field restriction may report having adequate vision to read regular print. Thus, it is important that the assessor not rely solely on the participant's near reading vision, but consider information about other visual functions, such as: distance visual acuity (e.g., recognizing people's faces, reading street signs, seeing television), visual fields, depth perception (difficulties ascending/descending stairs), etc. - Some assessors may benefit from specialized training on standardized reading measurements, so that they could utilize these instruments during the assessment, as appropriate.	- When choosing the appropriate response option, the assessor should consider the information available about other visual functions as well as reading up close. - It is important for the assessor to base their decision on the appropriate response option by using medical records as well, if available.

* *The alphanumeric code used in this table corresponds directly with items within the interRAI Community Health CHA Assessment Form and User's Manual Version 9.1*.

**Table 3:** Supplemental guidelines for completing section B (Vision and Hearing) within the Deafblind Supplement (DbS).

Specific item in the interRAI DbS (^*^)	**A. Suggestions for the assessor**	**B. Suggestions for the selection of the appropriate response option**
B2. Age of onset of vision loss	- The emphasis should be placed on the age at which the person began to lose their vision. The assessor should not assume that the date of diagnosis equals the date of onset of visual loss. In some visual diagnoses, like retinitis pigmentosa, a person may be diagnosed before they experience loss of vision. In some cases, a person may experience loss of vision for some time before they are diagnosed.	- If the respondent does not know the age of onset of their visual loss, do not assume that if the diagnosis is age-related, that the onset of visual loss occurred at age 65 or older. There are age-related visual conditions, such as age-related macular degeneration, that have an earlier onset.
B3. Classification of vision loss (a) Visual acuity range (b) Visual field diameter	- The assessor might also want to inquire whether the person is registered with a vision rehabilitation center. If so, they should try to access these medical records as they may contain low vision assessments that may not otherwise be available.	- None.
B4. Distance vision (Ability to see in adequate light with glasses or with other visual appliance normally used)	- The assessor should consider asking about the person's ability to see targets across the room other than faces. For instance, distance reading of street signs or watching television from across the room. The assessor should be aware that in some cases a person with visual loss may have difficulties with some activities (e.g., recognizing faces at a distance) and yet be able to identify objects from across the room.	- None
B7. Stability of vision condition	- Certain health conditions (e.g., diabetes, hypertension), can result in fluctuations in vision. For example, individuals with diabetes may notice a change in their vision when their blood sugar levels are not well-controlled. Other conditions are degenerative in nature (e.g., macular degeneration, glaucoma). Surgery (e.g., cataract/intraocular lens), among other medical interventions, could improve or adversely affect the stability of a person's vision. Be aware that in cases like these, the respondent may report fluctuations in vision that may make it difficult to select the appropriate response option. The longer the time frame, the more likely the fluctuations in vision has resulted in a lasting change from baseline.	- None.
B8. Diagnoses related to hearing loss	- Be aware that while the manual lists “conductive” and “sensori-neural,” there is a third type of hearing loss called “mixed” which is a combination of both conductive and sensorineural hearing loss. - It is not uncommon that a respondent will not be able to identify the medical term for their hearing loss diagnosis, but rather will report that the hearing loss is “age related.”	- None.
B9. Age of onset of hearing loss	- The emphasis should be placed on the age at which the person began to lose their hearing. The assessor should not assume that the date of diagnosis equals the date of onset of hearing loss. Certain hearing diagnoses (e.g., Usher syndrome) can result in a person being diagnosed before they experience a loss of hearing. Conversely, and, regardless of diagnosis, a person may experience a loss of hearing for some time before they are diagnosed.	- If the respondent does not know the age of hearing loss onset, do not assume that if the diagnosis is age-related, that the onset of visual loss occurred at age 65+ years. There are age-related hearing conditions, such as presbycusis, that can have an earlier onset.
B10. Location of sound	- The assessor should be aware of the hearing loss characteristics that are more likely to generate difficulties with sound location. The relevant characteristics include: degree of hearing loss; whether the loss is monaural (one ear) or binaural (both ears); and whether it is symmetrical (severity and shape of hearing loss are the same in each ear) or asymmetrical (each ear has a different severity and shape). Do not assume that if the person only wears one hearing aid, hearing is normal in the unaided ear. Often times, it is the unaided ear that has worse hearing. For example, a person with a normal hearing on one side, and a severe hearing loss on the other (whether sensorineural, mixed or conductive), will usually have difficulties with sound location. - How difficult it is to locate a sound varies depending on several listening conditions, including: the pitch or tone of the sound, how loud the sound is, and the sound target vs. background noise ratio. Thus, a respondent may be able to locate sounds during the interview, under optimal listening conditions, yet have difficulties locating sound in other real life situations.	- Do not assume that if the respondent locates sounds during the interview, that the person has no difficulties locating sounds. The assessor may want to ask the person about their difficulty in locating sounds under diverse situations of daily life (e.g., sounds of different tones against diverse levels of background noise). If the respondent reports having difficulties locating sounds under any scenario consider this information when choosing the appropriate response option.
	- The assessor may benefit from training in the use of different sound simulations (e.g., noise making kits) which could provide the assessor with another option to test sound location.	
B11. Alerting to different sounds	- The assessor should be aware that in both indoor and outdoor environments, responding to different sounds also has to do with the type of background noises present.	- None.
B12. Assistive devices or supports	- The assessor should note that the list is not exhaustive. Other commonly reported devices include: pocket talker (for hearing devices) and environmental alerting systems for the home (e.g., smoke, baby, telephone, doorbell), amplified telephone, infra-red TV amplification system for adaptive devices.	- None.

* *The alphanumeric code used in this table corresponds directly with items within the interRAI Community Health CHA Assessment Form and User's Manual Version 9.1*.

**Table 4:** Supplemental guidelines for completing interRAI DbS section C communication.

Specific item in the interRAI DbS (^*^)	**A. Suggestions for the assessor**	**B. Suggestions for the selection of the appropriate response option**
C1. Communication modes used daily	- The assessor should keep in mind that all of the communication modes could be used expressively or receptively. Thus, some modes may only be used for one purpose while others may be used for both purposes. For example, a person may use oral language for expression but not for reception, and use sign language for receptive and expressive purposes.	- None.
C2. One or more family members are able to communicate with person in person's preferred communication mode	- It is worthy to underscore the importance of this question. The mode of communication a person uses daily may not be their preferred mode, but rather the mode they must use to communicate with others in their environment. In such cases, the person may be far more skilled in their expressive language when using their preferred mode, compared to when using a different mode to enable communication with others. For example, a person may prefer to use sign language, but must use writing as no one in the environment has knowledge of sign language.	- None.

* *The alphanumeric code used in this table corresponds directly with items within the interRAI Community Health CHA Assessment Form and User's Manual Version 9.1*.

**Table 5:** Supplemental guidelines for completing interRAI DbS section I Orientation and Mobility (O&M).

Specific item in the interRAI DbS (^*^)	**A. Suggestions for the assessor**	**B. Suggestions for the selection of the appropriate response option**
I1. Orientation and mobility in daytime	- The manual focuses on orientation and mobility during daytime, knowing that for most people with vision and/or hearing loss, they will most likely have more difficulty at twilight and/or at night. This is because there are factors associated with night travel which are different from those present during the day (e.g., vehicle headlights, less visibility of objects/people, different sounds). - The assessor should be aware that different lighting conditions, both indoors and outdoors, can seriously impact a person's O&M performance. - It is important for the assessor to keep in mind that just because a person is familiar with an environment, this does not mean that they are sufficiently oriented, or has the necessary mobility skills to navigate the environment independently. For instance, a person may be oriented to their neighborhood, yet require accompaniment to go to the convenience store.	- None.
I2. Walking and trailing (person can differentiate among textures while walking [e.g., sidewalk, carpet] or while trailing [e.g., walls, doors])	- Because the domain asks for a person's ability to note changes in texture, the assessor may think that this ability is only related to perceiving with the skin. However, it could involve the information the person perceives through the use of mobility devices like a white cane or a wheelchair. It could also involve detecting changes in sound and smell.	- None.
I3. Travel (person feels safe traveling as a pedestrian [with assistive devices normally used])	- Because night travel presents different challenges to orientation and mobility than day travel, the assessor might consider asking about feelings of safe traveling after dark.	- None.

* *The alphanumeric code used in this table corresponds directly with items within the interRAI Community Health CHA Assessment Form and User's Manual Version 9.1*.

### Additional Questions for the Client That Will Help the Assessor Deepen Their Understanding of the Client's Sensory Status

It is recommended that assessors ask the client about multiple aspects of sensory loss which can help the assessor gain a more comprehensive understanding of the client's sensory status. More specifically, the CHA question regarding vision asks specifically about near vision. It is suggested that the assessor also inquire about the client's ability to see objects at a distance. Likewise, the CHA question for hearing focuses primarily on hearing speech. It is recommended that the assessor also ask about the client's ability to hear other sources of sound, such as processing concurrent multiple environmental sounds. The information generated from these additional questions can be added to the assessor's own clinical notes and contribute to their development of a service plan. Similarly, regarding the questions on vision and hearing within the DbS, assessors are encouraged to ask clients about additional aspects of their sensory loss. For example, the vision item on the DbS refers to the client's ability to see a face and/or object from across the room. We propose to also ask the client about other visual tasks, such as distance reading of street signs. Regarding the DbS question on hearing, we encourage the assessor to ask about the impact on the client's hearing ability of the sound's characteristics (e.g., pitch, volume), and levels of background noise.

### Additional Information That Will Help the Assessor Choose a Response to an Item on the Assessment

Assessor should not assume that if the client locates sounds during the interview that the person has no difficulty locating sounds. It is advisable to explore the client's ability in locating sounds under diverse situations of daily life (e.g., sounds of different tones against diverse levels of background noise), and to consider this information when choosing the appropriate response option.

### Supplementary Information on Sensory Impairment and Rehabilitation That Will Help the Assessor Administer the InterRAI CHA and DbS

Some suggestions stress how important it is to keep in mind that the client's hearing behavior during the interview is likely an incomplete picture of their overall hearing abilities. This is particularly true if the interview is carried out under optimal hearing conditions (e.g., in a quiet office room without competing noises, with an assessor that speaks slowly and provides non-verbal cues), which might mask the hearing problem a client would have in common daily life environments. As another example, Table 3 includes information about the typical age of onset of vision and hearing conditions and cautions that the assessor not make assumptions about the age of onset since some “age-related” conditions can have an “early” onset and thus do not necessarily entail an onset in late adulthood.

All specific suggestions are presented in the tables below. Note that each table is structured in the following way: Each row corresponds to the relevant question in the interRAI CHA and DbS. Column A “Suggestions for the Assessor” provides overall considerations for the assessor with the goal of supplementing the instructions in the interRAI user's manual. For example, this includes recommendations for performing the interview, with respect to its setting and questions, with the goal of obtaining the most comprehensive response to the item. This column also presents information specific to sensory loss that will help the assessor conduct the interview. Column B “Suggestions for Coding” includes considerations for how to choose a response in instances when definitions for the response options for the particular item would benefit from further elaboration, as well as information to clarify codes when their definitions could lead to misinterpretation. These recommendations were developed with the purpose of aiding assessors in administering the instrument to a group of older adults with sensory loss. Although we believe these suggestions will help assessors to gather information in an optimal way, the recommendations are ours alone and do not replace or change in any way the instructions and guidelines outlined within the interRAI CHA and DbS manual.

## Discussion

To our knowledge, this is the first study that provides specific recommendations for assessors when administering the interRAI CHA and DbS, the only existing measure validated and designed for use with adults that are living with combined vison and hearing loss. Our findings are particularly relevant given the complexities of assessing older adults with DSI since the combined sensory loss can have a profound influence on the person's ability to communicate with the assessor.

The supplemental guidelines we propose, used in tandem with the wealth of information, definitions and procedural instructions contained in the interRAI CHA user's manual, we hope will assist assessors to better understand sensory impairment and its importance in the assessment process. By following the proposed suggestions, we anticipate that assessors will deepen their knowledge on vision and hearing loss; more comprehensively understand the assessment's questions and response options; and optimize the completion of the assessment.

Included in Tables 2–5 are suggestions of topics to include in the training of future interRAI CHA and DbS assessors. Beyond these specific suggestions, we think that training would benefit overall from including trainers who have had exposure to sensory impairment information and thus can teach communication and assessment strategies tailored to clients with single or combined vision and hearing loss. We recognize that this recommendation is an ideal scenario that may not be feasible given clinical autonomy, time and resources, and hope that this study's suggestions can at least help future assessors gain some additional information on sensory loss, and trigger their curiosity for learning more about these topics.

Our recommendations are supported by several study strengths. Our sample is representative of older adults who are receiving sensory loss rehabilitation services at no cost to the client in an urban environment, in that participants were recruited from all three of Montreal's sensory rehabilitation centers (7). This study's assessor had significant clinical experience with adults that have VI, HH, or DSI, yet was novel to the interRAI CHA and DbS. This allowed for a qualitative evaluation of the utility of the assessment that could detect areas where additional information about sensory loss could help future assessor's administration of the instrument, as well as difficulties a novel assessor may encounter when first using the assessment.

This study also needs to be viewed and interpreted within certain limitations. All participants were receiving rehabilitation treatment for sensory loss, and thus, to varying degrees, had successfully navigated the health system to access these services. They were also reconciled with the loss of their vision/hearing and had acquired sensory compensation strategies and aids/devices. Additionally, participating in the study required several skills, such as: communicating verbally by phone, having the stamina for answering about 300 questions followed by a cognitive test. Thus, our findings may only apply to the most highly functioning older adults with VI, HH or DSI. Also, since most participants had acquired sensory impairment, our results may not apply to older persons with more severe or congenital sensory loss. Future studies should include recruitment and communication techniques that can reach older adults with all levels of sensory loss severity, and with a larger spread across the impairment spectrum (29). While the assessor's professional experience served to facilitate the qualitative evaluation of the assessment, it may have also minimized the level of difficulty involved in use of the instrument, particularly by those who are new assessors or have no disability-specific knowledge. For instance, whereas assessors in a previous study (14) had challenges with addressing items on mood and psychological well-being, the present study's assessor, who was an experienced social worker, did not experience these type of difficulties. Lastly, this study's results are limited since they are based on the experience of a single assessor, albeit one with extensive experience working with the population studied and who had some input from other professionals knowledgeable about sensory loss. Our results therefore need to be considered as an expert's opinion whom we methodological considered to be a “deviant case” (28).

This study reports concrete recommendations aimed to optimize the administration of the interRAI CHA and DbS, the only standardized interview instrument for adults that helps first-line health care providers to identify the needs, strengths and challenges for someone with DSI. Recommendations include additional questions that assessors can ask about a respondent's functional abilities, and information on sensory impairment and rehabilitation that will deepen assessors' understanding of vision and hearing loss. Together, these recommendations will help assessors more comprehensively understand the assessment's questions and response options, thereby allowing them to optimize the assessment process. In addition, we believe that that improved administration of the interRAI CHA and its DbS will be definition lead to more appropriate intervention and care. These findings are relevant given the increasing prevalence of DSI in the world's aging population. The detection and evaluation of DSI is of utmost importance because several aspects of health care delivery, and communication with health professionals, depend on it. The approach presented in this study will increase the awareness of sensory loss in older adults and its importance in assessment by first-line service providers.

## Data Availability Statement

The data utilized in this project cannot be made publicly available since data protection rules in Canada preclude them from being shared at the record level. The data were collected through a data sharing agreement with interRAI, the holder of the copyright to the CHA and Deafblind Supplement, and restrictions apply to the availability of these data. Requests to access the datasets should be directed to https://www.cihi.ca/en/data-inquiry-form.

## Ethics Statement

The studies involving human participants were reviewed and approved by Centre de recherche interdisciplinaire en réadaptation de Montréal métropolitain (CRIR-1018-1114). The patients/participants provided their written informed consent to participate in this study.

## Author Contributions

AU contributed to formal analysis, investigation, methodology, validation, writing-original draft, and writing-review and editing. CM contributed to data curation, formal analysis, investigation, methodology, resources, writing-original draft, and writing-review and editing. DG contributed to conceptualization, data curation, funding acquisition, investigation, project administration, resources, supervision, validation, and writing-review and editing. WW contributed to conceptualization, data curation, funding acquisition, investigation, methodology, project administration, resources, supervision, validation, and writing-review and editing. All authors agree to be accountable for the content of the work included in this study.

## Funding

This study was funded by the Canadian Consortium on Neurodegeneration and Aging (CCNA; http://ccna-ccnv.ca/en). The CCNA was supported by a grant from the Canadian Institutes of Health Research (Grant no. CNA-137794) (CIHR; http://www.cihr-irsc.gc.ca/e/193.html) with funding from several partners. Funding was also provided by the Foundation of the CRIR/Centre de réadaptation Lethbridge-Layton-Mackay du CIUSSS du Centre-Ouest-de-l'Île-de-Montréal, and the Fondation En Vue of the CRIR/Institut Nazareth et Louis-Braille du CISSS de la Montérégie-Centre. WW was supported by a Junior 2 *FRQS chercheur boursier* career award (#281454).

## Conflict of Interest

The authors declare that the research was conducted in the absence of any commercial or financial relationships that could be construed as a potential conflict of interest.

## Publisher's Note

All claims expressed in this article are solely those of the authors and do not necessarily represent those of their affiliated organizations, or those of the publisher, the editors and the reviewers. Any product that may be evaluated in this article, or claim that may be made by its manufacturer, is not guaranteed or endorsed by the publisher.
